# Editorial: The impact of abiotic stresses on agriculture: mitigation through climate smart strategies

**DOI:** 10.3389/fpls.2024.1391051

**Published:** 2024-05-14

**Authors:** Debojyoti Moulick, Shuvasish Choudhury, Marian Brestic, Akbar Hossain

**Affiliations:** ^1^ Department of Environmental Science, University of Kalyani, Kalyani, India; ^2^ Plant Stress Biology and Metabolomics Laboratory, Department of Life Science and Bioinformatics, Assam University, Silchar, India; ^3^ Institute of Plant and Environmental Sciences, Slovak University of Agriculture, Nitra, Slovakia; ^4^ Department of Botany and Plant Physiology, Czech University of Life Sciences Prague, Prague, Czechia; ^5^ Division of Soil Science, Bangladesh Wheat and Maize Research Institute, Dinajpur, Bangladesh

**Keywords:** abiotic stresses, changing climate, mitigation approaches, climate smart agriculture, food security

Components of agro-ecosystems, whether abiotic or biotic in nature, can be treated as key detrimental factors (for satisfactory growth and yield) or as a *stressor*(s) for crops/cropping systems when they are present below/above the optimum level either alone or in combination (s) (Choudhury and Moulick, 2022). Abiotic stress can be defined as hostile consequences imposed by abiotic component(s) on a crop in a particular agro-environment, resulting in a variety of responses ranging from alterations at the cellular level to gene expression, and metabolomics may have manifested in growth and yield reduction in crops (Choudhury et al., 2021; Zhang et al., 2022; Moulick et al., 2023). The most common abiotic stressors such as Salinity, toxic heavy metals/metalloids, flooding, drought, elevated ozone, carbon dioxide, methane, nitrous oxide, lack of nutrients, extreme temperatures, and nanoparticles pose serious challenges to achieving global food security.

Climate-smart agriculture is a polymathic approach to altering and redesigning the agro-ecosystems to support global food security under the new realities of climate change. Researchers from different parts of the world have contributed their research findings to this topic. Among the published content on this topic, research on both food and forage crops can be found, highlighting that the consequences of salinity, drought, minimizing greenhouse gas emissions, and heat stress are predominant.

In an era of severe global climatic fluctuations, a series of cellular and biochemical manifestations lead to a burst of reactive oxygen species (ROS) upon encountering a stressor, which is subsequently manifested as a reduction in pigments (Shariatipour et al.), the ratio of Fv to Fm, i.e., the chlorophyll fluorescence parameter (Elfanah et al.), transcriptomic profiles, signaling behavior (Zhao et al.), and alterations in the gene expression of aqua porin and heat shock protein (Hongal et al.). To achieve resilience to abiotic stressors, assessing the intensity of damage caused by stressors on a given crop is vital. Efficacy of hyperspectral reflectance and agro-physiological traits. In addition to its robust and statistically sound experimental design, this approach has been proven to be valuable (Elfanah et al.). Using tolerance indices, Alam et al. identified suitable salt-tolerant onion genotypes.

Melash et al. documented the efficacy of nutrient management on the qualitative aspect of caryopsis and yield attributes of durum wheat under the regime of changing climatic conditions. The article concluded that custom-made (case-by-case) nutrient management strategies, agronomists, breeders, and farmers can play a vital role in durum wheat production, safeguarding food security. N_2_O and CH_4_ emissions can be effectively reduced in high-yielding transgenic rice with partial aerenchyma by immersing root exudates, which provide substrates for GHGs. Observations made by Iqbal et al. have demonstrated the ability of plant breeders/biotechnologists to serve the goal of sustainable development. In another interesting study, the authors reported that supplementing SiNPs can effectively mitigate salinity stress in lemongrass (*Cymbopogon flexuosus* (Nees ex Steud.) Wats. (Mukarram et al.). Shi et al. provided important insights into the genes and mechanisms underlying the resilience of crop plants to Si-induced low-Fe stress. The authors observed that Si supplementation under Fe-deficient conditions amplified Fe supply to the leaves and roots of tomato plants. With gradual progress in the sowing date, a delayed trend in tiller initiation and a prolongation of later growth phases were observed in the studied wheat varieties cultivated in the Indo-Gangetic Plains. Sattar et al. concluded that the date of sowing and the environment are crucial factors in determining phenology and yield. Qu et al. reported that drought can significantly decrease the carbon (22.7%), nitrogen (21.2%), and phosphorus (21.6%) contents of microbial biomass and the activity levels of enzymes such as β-1,4-glucosidase (26.8%) and acid phosphatase (16.0%) under drought conditions in terrestrial ecosystems.

There is always room for improvement in research, especially in the agro-environmental domain, where multiple factors are operating at different scales. An urgent need to suggest a particular remedial measure is the validation of findings in multiple environments along with multiple crop/cropping systems to deepen our understanding of how a particular variety (existing/newly developed) interacts with the environment and management. Moreover, under constantly fluctuating climates, research on soil conditions should focus on validating the United Nations SDGs. This particular Research Topic provides a platform to highlight some interesting findings; however, there is an urgent need for sustainable and environmentally friendly interventions, such as those related to the molecular response of plants and stressor interactions, the potential for genetic engineering, wild relatives of crops, seed priming, water and nutrient management, and postharvest quality assessment (e.g., eating and cooking attributes), which should be prioritized in the near future (Hossain et al., 2022; Hazra et al., 2023; Moulick et al., 2024). Finally, due emphasis should be given to strategic research to maintain the flow of information/feedback for both basic and applied research.

## Author contributions

DM: Conceptualization, Writing – original draft, Writing – review & editing. SC: Conceptualization, Writing – original draft, Writing – review & editing. MB: Conceptualization, Writing – original draft, Writing – review & editing. AH: Conceptualization, Writing – original draft, Writing – review & editing.
